# The relationship between social anxiety and self-injury of junior high school students: Mediation by intolerance of uncertainty and moderation by self-esteem

**DOI:** 10.3389/fpubh.2023.1046729

**Published:** 2023-03-09

**Authors:** Zhendong Yao, Lu Pang, Jin Xie, Seqin Shi, Min Ouyang

**Affiliations:** ^1^Normal College, Hunan University of Arts and Science, Changde, China; ^2^School of Preschool Education, Hunan College for Preschool Education, Changde, China; ^3^Mental Health Service Center, Huanghuai University, Zhumadian, China; ^4^No. 1 Middle School of Loudi, Loudi, China; ^5^Students Affairs Department, Xiangnan University, Chenzhou, China; ^6^College of Educational Science and Law, Xiangnan University, Chenzhou, China

**Keywords:** junior high school student, self-injury, social anxiety, intolerance of uncertainty, self-esteem

## Abstract

**Objective:**

The problem of adolescents' self-injury has gradually attracted social attention, however, a lack of research exists on the internal mechanism between social anxiety and self-injury. This study explored the relationship between social anxiety and self-injury in Chinese junior high school students.

**Method:**

An adolescent self-injury questionnaire, social anxiety scale, intolerance of uncertainty questionnaire and self-injury questionnaire were used to survey 614 junior high school students.

**Results:**

The results showed that: (1) social anxiety had a significant positive predictive effect on self-injury; (2) intolerance of uncertainty had a significant mediating effect between social anxiety and self-injury; and (3) self-esteem had a significant moderating effect on the mediating effect of intolerance of uncertainty.

**Conclusion:**

The study suggested that social anxiety in junior high school students has an impact on self-injury through mediation of intolerance of uncertainty and modulation of self-esteem.

## Introduction

Adolescence, often referred to as the “storm period,” includes the junior high school stage, an important period for individual physical and mental development. The psychological characteristics of junior high school students have two aspects: autonomy and dependence. They not only want to be independent, but also want to be helped by others (1). The way teenagers express their emotions changes transitions from externalism to concealment, and sometimes they hide their true emotions without expressing themselves (2). For this reason, many junior high school students often lose their psychological balance, causing various problems arise, such as self-injury, to arise.

### The relationship between social anxiety and self-injury

Non-suicidal self-injury refers to the behavior of intentionally and repeatedly hurting one's body without suicidal intention, which is not accepted by society and will not lead to death (3). In China, teenagers comprise a high-risk group for self-injury (4). A significant public health problem, the incidence of self-injury among adolescents is as high as 30% (5). Although it has a certain universality, it is often a hidden problem (6). These venting behaviors do not always occur in public. Some behaviors vent in unreasonable and potentially negative ways when the individual is alone, prompting negative coping styles that may increase the risk of non-suicidal self-injury (7). What is the cause of self-injury in individuals? According to the system model, self-injury is a symptom manifestation of environmental dysfunction. Environmental influences may unintentionally enhance self-injury because self-injurious can transfer dysfunction. Additionally, individuals can use self-injurious behavior to obtain external attention (8). The core feature of self-injury is an emotional management disorder (9). In summary, social anxiety may be the cause of self-injury.

Social anxiety refers to the psychological phenomenon of nervousness, fear, and embarrassment in the process of normal interpersonal interaction with others (10). Individual social anxiety begins in adolescence (11), because compared with the previous primary school period, the pupils' compliance with parental arrangements and patience with teachers' instruction begin to gradually shift to the pursuit of “self.” Studies have shown that self-awareness has some effect on social anxiety (12). Family and school are the main environments where junior high school students communicate placing the focus on the family members, classmates, and teachers. The junior high school period is a time when students' self-consciousness takes a great leap (13). Studies have proven the existence of a relationship between anxiety and self-injury (14). Further, research has indicated that specific extrinsic actions are necessary for people with intrinsic anxiety, because, they can express their feelings through extrinsic self-injury as an outlet (15). Social anxiety is a type of anxiety that corresponds with the above research results. It has been suggested that individuals with a higher degree of social anxiety can interpret facial expressions as negative (16). Moreover, the emotional instability of people with anxiety disorders may lead to non-suicidal self-injury (17). Although previous studies have demonstrated the influence of social anxiety on self-injury, few studies have explored the underlying mechanism. To prevent self-injury behavior and provide effective intervention measures for the self-injury of junior high school students, we examined a moderated mediation model to uncover the possible mechanisms underlying this relationship.

### The mediating role of intolerance of uncertainty

Prior research indicates that individuals with social anxiety have negative processing bias toward positive stimulation (18), and tend to often be emotionally unstable, impatient and impulsive (19). Such individuals experience more discomfort, which leads to poor or even little to no tolerance of uncertain events. Intolerance of uncertainty refers to the aversive response of an individual due to their inability to perceive significant or sufficient information, sustained by uncertainty perception (20). Intolerance of uncertainty is especially closely related to social anxiety (21). The inability to tolerate uncertainty may be an important component of anxiety (22) and may be one of the key features of anxiety disorder referrals (23). Therefore, it is logical reason that social anxiety can be positively related to intolerance of uncertainty.

Moreover, intolerance of uncertainty not only affects one's physical and mental health, but also one's decision-making and problem solving ability. Individuals with high intolerance of uncertainty are more biased toward the occurrence of potentially adverse events (24). In daily diagnoses, intolerance of uncertainty can be a powerful indicator in examining the rehabilitation of anxiety disorders (25). Because of the close relationship between negative emotions and self-injury, negative emotions are often used as the primary indicator to assess self-injury functioning (26). Junior high school students are faced with increasing learning tasks and complicated interpersonal communication. During this period, when social anxiety is common, students often do not know how to relieve their inner discomfort, prompting them to inflict non-suicidal self-injury to rescue themselves. Based on the above, this paper put forward the assumption:

**Hypothesis 1**. Social anxiety is positively related to intolerance of uncertainty, which in turn would be positively related to self-injury.

### The moderating role of self-esteem

Although social anxiety may increase the risk of self-injury through the mediating role of intolerance of uncertainty, not all individuals with high social anxiety experience higher levels of intolerance of uncertainty and self-injury. Therefore, it is necessary to explore the potential moderating variables between social anxiety and self-injury. The present study tests the hypothesis that the links between social anxiety and self-injury are moderated by self-esteem.

According to the hypothesis regarding the relief of anxiety, self-esteem is a natural buffer to cope with anxiety. Social maladjustment causes teenagers to lack effective countermeasures when facing uncertain external events (27), making young people unable to tolerate increased levels of uncertainty. It is believed that the flexible space provided by the self-regulation mechanism of self-esteem can help people alleviate social anxiety (28). Self-esteem is an important variable among the indicators of psychosocial adjustment of individuals. It refers to a person's cognition, attitude and view of one's own personal ability or personal value (29), and plays an important role in monitoring individual interpersonal relationships (30). Low self-esteem may be a psychological variable related to self-injury (31). The higher the level of self-esteem, the lower the risk of anxiety and the less possibility of anxiety related behaviors (32). For instance, left-behind children with higher levels of self-esteem experience a weaker negative impact on self-injury by stressful life events than left-behind children with lower levels of self-esteem (33). According to the above literature, we put forward the following assumptions:

**Hypothesis 2**. Self-esteem moderates the relationships between social anxiety and self-injury.

### The present study

**Figure 1:**
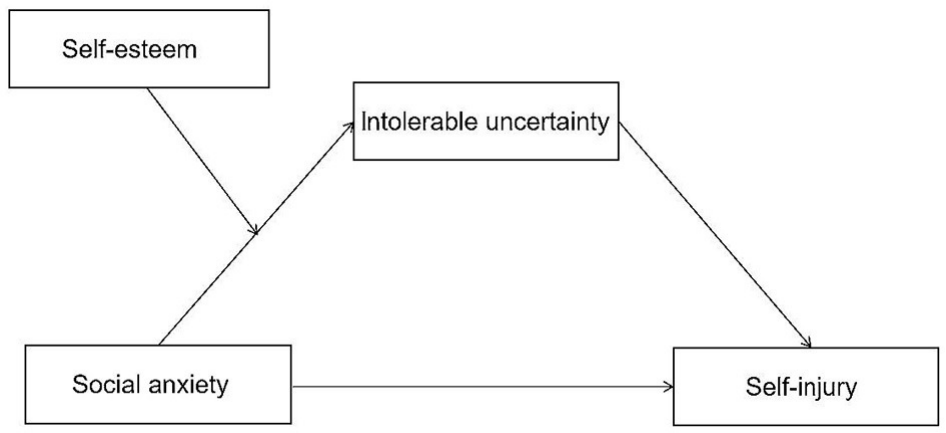
The proposed moderated mediation model.

The objectives of this study were as follows: (a) to examine the mediating role of intolerance of uncertainty between social anxiety and self-injury, and (b) to examine whether the relational pathway between social anxiety and self-injury is moderated by self-esteem. This study conducted a moderated mediation analysis to test these hypotheses. Therefore, Figure 1 presents a moderated mediation model.

## Methods

### Participants

Through convenient sampling, four junior high schools of Hunan Province, China were investigated. A total of 700 questionnaires were distributed and 614 valid questionnaires were returned, with an effective rate of 87.71%. Upon analysis of the sample population, 263 (42.80%) were males and 351 (57.20%) were females. The mean age of the participants was 13.91 years (*SD* = 1.01), with a range of 13–15 years. Regarding grade level, 205 (33.40%) were in the first grade, 223 (36.30%) in the second grade, and 223 (36.30%) in the third grade.

### Measures

#### Social anxiety scale

The Social Anxiety Scale (SAS) is a subscale derived from the self-awareness scale compiled by Fenigstein et al. (34). The Social Anxiety Scale is one of the subscales. The Chinese version of the Social Anxiety Scale was selected from the Mental Health Rating Scale Manual edited by Wang et al. (35), which has a total of six question items, such as “I often speak and feel anxious in front of the population.” In this study, a 5-point Likert scale was used (1 = very inconsistent, 2 = somewhat inconsistent, 3 = unclear, 4 = somewhat consistent, and 5 = extremely consistent), indicating the degree of social anxiety. In this study, Cronbach's α of the Social Anxiety Scale was 0.74.

#### Adolescent self-injury questionnaire

This paper selects from Feng (36), which was based upon the questionnaire developed by Zheng and the self-injurious behavior scale developed by Graze, a revised localized and standardized scale, which has good psychometric indicators. The questionnaire consists of 18 items, such as “deliberately grasping oneself violently and achieving the degree of scarring or bleeding,” and 1 open-ended item (In this study, the number of people who filled in this question was less, so only 18 closed-ended items were counted). Among them, the options for the number of self-injury occurrences are 0, 1, 2 – 4, and more than 5; the options for the degree of self-injury to the body are: none, mild, moderate, severe, and very severe. In this study, the Cronbach's alpha of the self-injury questionnaire was 0.95.

#### Intolerance of uncertainty scale

This item was selected from the Chinese version of the Intolerable Uncertainty Scale (simplified version) revised by Lijuan et al. (37). This questionnaire consists of 12 question items, such as “When I am not sure, I cannot do well,” which are divided into three dimensions, anticipatory behavior, inhibitory behavior, and anticipatory emotion. Responses were given on a 5 point Likert scale: 1 = completely inconsistent, 2 = somewhat inconsistent, 3 = basically in conformity, 4 = very in conformity and 5 = completely in conformity. In this study, the Cronbach's alpha of the self-injury questionnaire was 0.80.

#### Self-esteem scale

The Self-Esteem Scale (SES) was developed by Rosenberg, and is used to measure teenagers' overall feelings about self-worth and self-acceptance (38). The scale includes 10 items in the scale, such as “generally speaking, I am satisfied with myself.” A 4 point Likert scale was used: very good agreement = 4, conformity = 3, non-conformity = 2, and extreme non-conformity = 1. In this study, the Cronbach's α of self-injury questionnaire was 0.84.

### Data analysis

In this study, IBM SPSS Statistics 26.0 was used to analyze the collected data, Hayes' PROCESS macro program (2012) was used to analyze the mediating effect, and bootstrap method was used to test the significance of regression coefficient (39). The sample distribution was reconstructed by random sampling with replacement. In this study, a total of 5,000 samples were constructed, each with a sample size of 614. From this, the standard errors and confidence intervals of the parameter estimation were calculated. If the confidence interval does not include zero, it means the results are significant.

## Results

### Preliminary statistics

There may be common method bias in the data of the self-assessment questionnaire. In this study, the Harman's single-factor test was used to test the effect of procedural control (40). The four variables of social anxiety, intolerable of uncertainty, self-esteem, and self-injury were combined and texted using IBM SPSS 26.0. The results indicated 11 factors with eigenvalues >1.0, and the first common factor is 14.39% of the variance, which was far less than the critical standard of 40%. Therefore, common method bias was unlikely in this study.

The results of the correlation analysis of social anxiety, intolerance of uncertainty, self-esteem and self-injury are presented in Table 1. There were significant positive correlations between all variables, which were consistent with theory and previous research. Social anxiety was positively correlated with intolerance of uncertainty (*r* = 0.50, *p* < 0.01) and self-injury (*r* = 0.18, *p* < 0.01). Additionally, intolerance of uncertainty was positively correlated with self-injury (*r* = 0.18, *p* < 0.01). Moreover, self-esteem was related inversely related to the social anxiety (*r* = −0.30, *p* < 0.01) and intolerance of uncertainty (*r* = −0.30, *p* < 0.01) and self-injury (*r* = −0.15, *p* < 0.01).

**Table 1:** Descriptive statistics and correlations between study variables.

**Variables**	***M***	***SD***	**1**	**2**	**3**	**4**
1. Social anxiety	18.50	4.57	1			
2. Intolerance of uncertainty	32.79	7.31	0.50^**^	1		
3. Self-esteem	28.00	4.87	−0.30^**^	−0.30^**^	1	
4.Self-injury	6.58	14.31	0.18^**^	0.18^**^	−0.15^**^	1

** *P* < 0.01.

### Testing for mediation effect

Considering the advantages of applying the Bootstrap method in testing the mediating effect (41), the PROCESS PROCEDURE for SPSS developed by Hayes was used to test the mediating effect (model 4). The results showed that the mediating effect value is *F*_(1,614)_ = 50.57, *P* < 0.001, *R*^2^ = 0.25, the mediating effect value is 0.061, and the confidence interval of 95% is [0.020, 0.11], indicating that the mediating effect is significant, accounting for 57.49% of the total effect and 61.00% of the direct effect. Therefore, Hypothesis 1 was supported.

### Test of the moderated mediation model

According to the research theory of Muller et al. (42) and others, this study used three regression equation tests to check the mediation models with regulation one by one. Equation 1 examined the moderating effect of self-esteem on social anxiety and self-injury of junior high school students. Equation 2 examined the moderating effect of self-esteem on the relationship between social anxiety and intolerance of uncertainty. Equation 3 examined the moderating effect of self-esteem on the relationship between intolerance of uncertainty and self-injury, and the moderating effect of social anxiety and self-injury participation. Among the three regression equations, any significant moderating effect can show that self-esteem plays a moderating role in the relationship between social anxiety and self-injury of junior high school students.

In this study, independent variables, mediating variables, and moderating variables were all standardized, and gender, grade, and age were controlled by means of hierarchical regression (see results in Table 2). In Equation 1, self-esteem was significant for the relationship between social anxiety and self-injury in junior high school students (β = −0.082, *P* = 0.023). In Equation 2 self-esteem was significant for the relationship between social anxiety and intolerance of uncertainty in junior high school students (β = −0.63, *P* = 0.047). In Equation 3, self-esteem modulated the effects of social anxiety and self-injury in junior high school students and the participation effects of social anxiety and self-injury in junior high school students (β = −0.048, *P* = 0.16). In summary, self-esteem has a moderating effect on the social anxiety of intolerance of uncertainty. Therefore, Hypothesis 2 was supported.

**Table 2:** The moderating effect of self-esteem qon the mediation of intolerance of uncertainty.

	**Model 1 (Self-injury)**		**Model 2 (Intolerance of uncertainty)**		**Model 3 (Self-injury)**	
	**β**	***t***	**β**	***t***	**β**	***t***
Gender	0.023	0.29	0.038	0.53	0.024	0.30
Age	0.14	2.26^*^	0.096	1.74	0.13	2.02^*^
Grade	−0.30	−3.86^**^	−0.080	−1.16	−0.29	−3.73^**^
Social anxiety	0.14	3.41^**^	0.45	12.43^**^	0.090	1.95
Self-esteem	−0.088	−2.10	−0.16	−4.25^**^	−0.070	−1.63
Social anxiety × Self-esteem	−0.082	−2.28^*^	−0.063	−2.00^*^	−0.047	−1.40
Intolerance of uncertainty					0.10	2.17^*^
Intolerance of uncertainty × Self-esteem					−0.048	−1.41

* *P* < 0.05 and

** *P* < 0.01.

**Figure 2:**
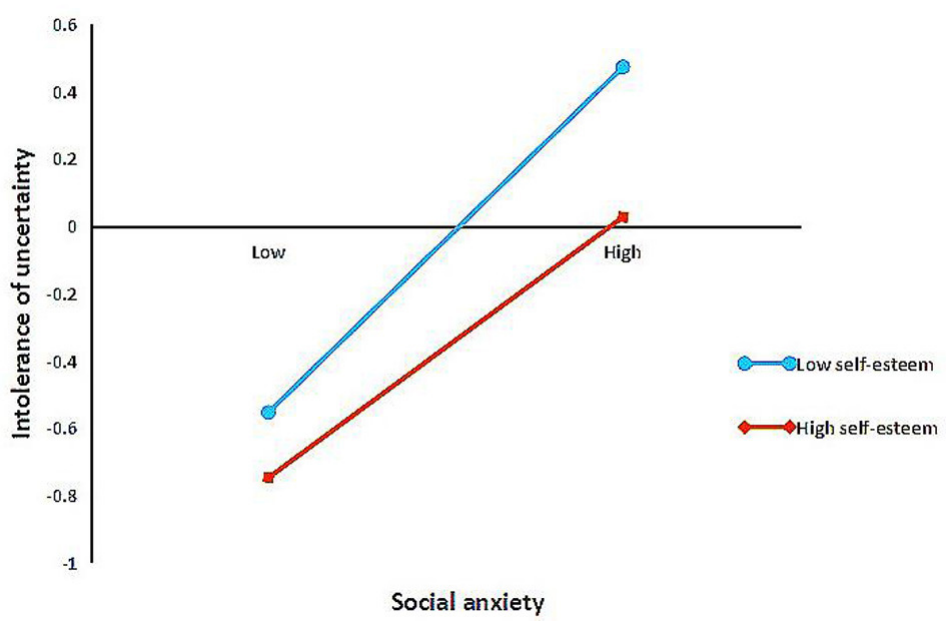
Association between social anxiety and self-injury at higher and lower levels of self-esteem.

In order to analyze the moderating effect of self-esteem on the mediating effect of intolerance of uncertainty more clearly, a simple slope test was conducted on the interaction, as demonstrated in Figure 2. The specific method was as follows: based upon their score of self-esteem, the subjects are divided into high (Z ≥ 1 SD and low (Z ≤ - 1 SD) groups, to investigate the intolerance of uncertainty on different levels of self-esteem among junior middle school students, and to draw a simple effect analysis diagram. The results demonstrate that when the self-esteem level of junior high school students is at a low level, social anxiety has a significant negative predictive effect on intolerance of uncertainty (β = 0.52, *p* < 0.00). When the self-esteem level of junior high school students is high, the influence of social anxiety on intolerable of uncertainty is reduced (β = 0.39, *p* < 0.00).

## Discussion

### Social anxiety and self-injury of junior high school students

The phenomenon of self-injury is very common among adolescents, while it may also be in an ascending state (43). According to Mathew et al. (44) research, self-injured individuals scored higher on anxiety assessment. According to Benjet et al. (45), the occurrence of anxiety is associated with that of self-injury. That, anxiety may become a negative emotion that causes self-injury (46). What is the reason for the social anxiety of junior high school students? Prior research has indicated that social anxiety derives from self-awareness. If individuals are unaware, it is difficult for them to generate anxiety. Feelings of anxiety may lead to a decrease in the individual's motivation in social competition and the emergence of avoidance strategies (47).

Why do anxious individuals tend to self-injure? According to Hasking et al. (48) research, the emotional instability of patients with anxiety disorders may lead to self-injury and the client's impulsivity is very strong, leading to non-suicidal self-injury. For junior high school students, the emergence of social anxiety is related to their developmental stage—e.g., the rapid development of the adolescent physiology of boys and girls leads to dramatic changes in their respective psychology, adding complex factors to their processing of interpersonal relationships. In addition, due to the increase of academic pressure among junior high school students, they must experience the process of navigating self-study, personal life, and getting along with others. Coordinating the relationships with multiple people can be a challenge, thus, dysregulated symptoms can emerge among various social contacts.

In this study, junior high school students' social anxiety has a significant positive predictive effect on self-injury. The results of this study are consistent with the above theory. It is evident that individuals prone to self-injury exhibit higher scores on anxiety items in self-assessment; and their anxiety experience is clear. Thus, junior high school students are more prone to self-injury due to the painful experience of social anxiety. Just as previous studies have indicated, interpersonal relationships are an important driving force for the development of adolescent self-injury (49). Regarding individuals within in a certain social structure who have self-injured, anxiety will cause them to vent through self-injury (50).

### The mediating role of intolerance of uncertainty

Upon further examination, this study found that the mediating effect of intolerance of uncertainty between social anxiety and self-injury of junior high school students is significant. Per prior research, social anxiety is related to intolerance of uncertainty (51). Moreover, there is a significant positive correlation between social anxiety and intolerance of uncertainty (52). The results of this study are consistent with those of previous studies.

Intolerance of uncertainty reflects an individual's perception of uncertainty, and, is not related to the real situation or the threat (53). Therefore, intolerance of uncertainty reflects the individual's inner psychological security. Individuals with high intolerance of uncertainty tend to cognitively process uncertain or vague situations in a negative way, so they are more likely to produce negative emotions (54). These difficulties in regulating emotions may lead them to hurt themselves (55). Previous studies on adolescent self-injury also corresponded to the above research. Adolescents experencing non-suicidal self-injury have difficulty with emotional regulation (56). Based on the above logic, the treatment of anxiety and intolerance of uncertainty may help to resolve issues with social problems (57), such as self-injury.

### The moderating role of self-esteem

The results showed that intolerance of uncertainty was a mediating variable between social anxiety and self-injury in junior high school students, and that self-esteem was a moderating variable between social anxiety and intolerance of uncertainty in junior high school students. The results of this study are consistent with the prior theoretical logic. Per prior research, improving the self-esteem of Chinese emerging adults (58) can help reduce their social anxiety (59). Why does self-esteem help reduce anxiety? According to the theory of fear management, the flexible space provided by the self-regulation mechanism of self-esteem can help individuals effectively relieve anxiety (60). Therefore, when an individual's self-esteem is enhanced, anxiety is alleviated, and more effective actions are under taken to help the individual to better return to a balanced life. In other words, self-esteem may be one of the protective factors of junior high school students. These findings are consistent with the study's hypothesis 2.

Further analysis of the findings indicate that individuals with higher self-esteem can reduce the negative influence of anxiety on their psychology and behavior (32). Conversely, individuals with low self-esteem demonstrate higher intolerance of uncertainty (61). Consequently, this study confirms that self-esteem is an important protective factor that can reduce the negative impact of social anxiety and ameliorate the intolerance of uncertainty.

### Limitations and implications

There are several limitations in this study. First, this study used self-reported methods to collect data. Although, there is no serious collinearity in this study, future studies should collect data in a variety of ways, such as collecting other comments, and through observation and interview methods. Second, a cross-sectional research design was used to detect the relationship between social anxiety and self-injury in junior high school students. However, cross-sectional studies do not easily reveal causal relationships, thus, future studies could use experimental or longitudinal methods to examine them in depth. Third, the sample selected for the study was only a subset of junior high school students in Hunan Province, China, which lacked representativeness. The study needs to further recruit a different representative sample. This study only explored the moderating role of self-esteem between social anxiety and intolerance of uncertainty, and did not explore the moderating role of self-esteem between social anxiety and self-injury. Future research could simultaneously examine the moderating role of the direct and indirect between social anxiety and self-injury.

Although there are limitations in the research, the results of the study have implications for theory and practice. First, this study expanded upon previous research by confirming the mediating role of intolerance of uncertainty and the moderating role of self-esteem. This could contribute to a better understanding of how and when social anxiety can be related to self-injury. Second, our study demonstrated that self-esteem played a protective role to reduce negative influence. This means that it is meaningful to improve junior high school students' self-esteem, thereby reducing the incidence rate of self-injury. Third, we should intentionally guide junior high school students to develop positive self-esteem, so as to enhance their ability to resist negative stimuli. School education can be enlightened as follows: in the prevention of self-injury in junior high school students, it can improve the self-esteem level of individuals by ameliorating their social anxiety and intolerance of uncertainty, and through mental health education activities, such as a second classroom and other forms, to achieve the expected results (62).

## Conclusion

This study revealed that self-injury served as a potential mechanism by which serious social anxiety were associated with self-injury in a sample of Chinese junior high school students. Moreover, the association between social anxiety and intolerance of uncertainty had a more significant impact for junior high school students with a lower level of self-esteem compared with those with a higher level of self-esteem.

## Data availability statement

The raw data supporting the conclusions of this article will be made available by the authors, without undue reservation.

## Author contributions

ZY and MO designed the study protocol, provided financial support, as well as guided the first draft of the manuscript, provided guidance on the overall design of the study, and the revision of the manuscript. JX and LP performed the statistical analysis, drafted the manuscript, completed the literature review, and participated in the study design and interpretation analysis. SS guided the statistical analysis, interpretation of the results, edited the final manuscript, and provided guides in the process of revision. All authors contributed, read, and approved the submitted version.
